# Hyaluronic Acid: A Review of the Drug Delivery Capabilities of This Naturally Occurring Polysaccharide

**DOI:** 10.3390/polym14173442

**Published:** 2022-08-23

**Authors:** Ciara Buckley, Emma J. Murphy, Therese R. Montgomery, Ian Major

**Affiliations:** 1 PRISM Research Institute, Technological University of the Shannon, N37 HD68 Athlone, Ireland; 2 Biosciences Research Institute, Technological University of the Shannon, V94 EC5T Limerick, Ireland; 3 LIFE Research Institute, Technological University of the Shannon, V94 EC5T Limerick, Ireland; 4 School of Science and Computing, Atlantic Technological University, H91 T8NW Galway, Ireland

**Keywords:** naturally-occurring polymers, polysaccharide, immunotherapies, bioactives, heteropolysaccharides, drug-delivery

## Abstract

The inclusion of physiologically active molecules into a naturally occurring polymer matrix can improve the degradation, absorption, and release profile of the drug, thus boosting the therapeutic impact and potentially even reducing the frequency of administration. The human body produces significant amounts of polysaccharide hyaluronic acid, which boasts exceptional biocompatibility, biodegradability, and one-of-a-kind physicochemical features. In this review, we will examine the clinical trials currently utilizing hyaluronic acid and address the bright future of this versatile polymer, as well as summarize the numerous applications of hyaluronic acid in drug delivery and immunomodulation.

## 1. Introduction

Hyaluronic acid (HA) is a naturally occurring mucopolysaccharide that belongs to a group of heteropolysaccharides referred to as glycosaminoglycans (GAGs) [1,2,3]. Mucopolysaccharides are long chain sugar molecules commonly found in mucus or joint fluid in the body. Endogenous HA is found throughout the human body in the vitreous humour, joints, umbilical cord, connective tissue, and skin. Naturally, occurring HA is commonly isolated from sources such as rooster comb for industrial applications requiring an animal source. However, it can also be synthesized using biotechnological processes and recombinant DNA technologies in bacterial expression systems such as Lactococcus. A species of bacteria that can naturally produce HA and therefore HA can be isolated from, is Streptococcus. However, Streptococcus is a pathogen that produces several endotoxins, rendering end-stage product isolation difficult. As a result, other approaches such as cell-free HA synthesis or genetic engineering of microorganisms that do not create endotoxins have been developed [4]. This freely accessible natural polysaccharide has a wide range of applications due to its unique physicochemical and bioactive properties, which will be explored in depth throughout this review. Furthermore, HA is biocompatible and biodegradable, making it a safe biomaterial for biomedical applications such as biomedical engineering, as well as finding applications in the cosmetics industry, wound healing, and drug delivery [5,6,7]. Through this review, we will first discuss the discovery of HA, followed by an in-depth analysis of its physicochemical and bioactive properties, as well as its synthesis and degradation, functionalization, and applications in drug delivery, wound healing, and cancer therapeutics. In conclusion, we will review the potentially bright future of HA, with a particular emphasis on ongoing as well as forthcoming clinical trials.

## 2. Discovery of HA

Karl Meyer and his colleague John Palmer discovered HA in 1934 when they isolated a previously unknown material from the bovine vitreous body. They named this novel substance hyaluronic acid after discovering that it included two sugar molecules, one of which was uronic acid. The “hyal” portion came from the word hyaloid which refers to the vitreous body hence the combination was called hyaloid + uronic acid or hyaluronic acid [8,9]. This previously unknown substance would go on to become one of the most intriguing and extensively studied naturally occurring human polymers. HA was not used commercially until 1942, when Endre Balazs submitted a patent to replace egg whites with HA in baked goods [9,10]. However, it is not known if this patent was granted and the authors have not been able to find a reference to HA being used as a suitable alternative to egg white in baking. Subsequently, in the late 1950s, HA found its way into medical applications when it was used to replace the vitreous humour of the human eye.

Although it was first identified as an acid and is frequently referred to as hyaluronic acid, HA acts more similar to the salt, sodium hyaluronate, under physiological conditions. Endre Balazs coined the name hyaluronan in 1986 [11], thereby capturing the different forms HA might take while conforming to the international polysaccharide nomenclature- the acid and the salt.

## 3. Physicochemical Properties of HA

### 3.1. Structure

**Figure 1:**
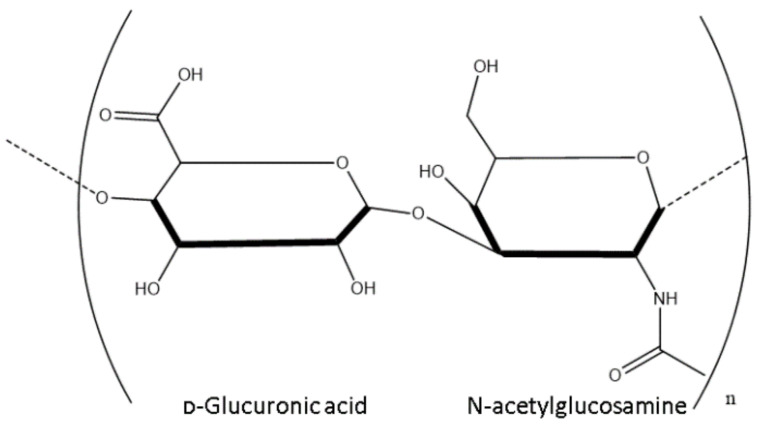
Structure of a disaccharide of HA.

HA is an anionic polymer consisting of disaccharides of D-glucuronic acid and N-acetyl-D-glucosamine, which are linked by β (1, 4) and β (1, 3) glycosidic bonds as shown in Figure 1 below.

HA is renowned for its unusual viscoelastic properties, due to the interaction between chains of hydrogen bonds. The HA macromolecule is best represented as a hydrated spherical. In its most elongated conformation, HA exhibits its highest viscosity in an aqueous solution. As the concentration of HA in aqueous solution increases, so does the viscosity due to chain weaving and the formation of 3-dimensional matrices. This is the basis of gelation, however, salts can be added as viscosity modifiers to facilitate the use of high-concentration solutions, as demonstrated by Selyanin et al. [7].

HA is a polymer that is extremely hydrophilic. Each disaccharide contains a carboxylic acid component that dissociates at physiological pH, enhancing the polyanionic nature of the polysaccharide. Because of this polyanionic behavior, several metal ions can be coupled to the hydration shell, resulting in a 1000-fold increase in volume and the formation of 1000 weakly packed hydrated matrices [12]. This is the basis of HA’s physiological functions, such as its rheological characteristics, elasticity, wound healing capacity, and cell lubrication, and it also explains HA’s involvement as a structural component of the ECM [13].

### 3.2. Molecular Weight

The biological function of HA is mainly dependent on the polymer’s molecular weight (MW) [14,15,16,17]. Table 1 below summarises the average molecular weights of HA from different areas of the human body, however, this is not an exhaustive list as HA is ubiquitous throughout the body. While it typically exists as a high molecular weight polymer, of over 106 Daltons (Da) or 1000 Kilodaltons (kDa), it can be cleaved by an enzyme, hyaluronidase, in the body to obtain molecules of much lower MWs. The biological functions include control of tissue hydration, supramolecular assembly of proteoglycans in the extracellular matrix, and multiple roles in receptor-mediated cell detachment, mitosis, and migration [18,19].

**Figure 2:**
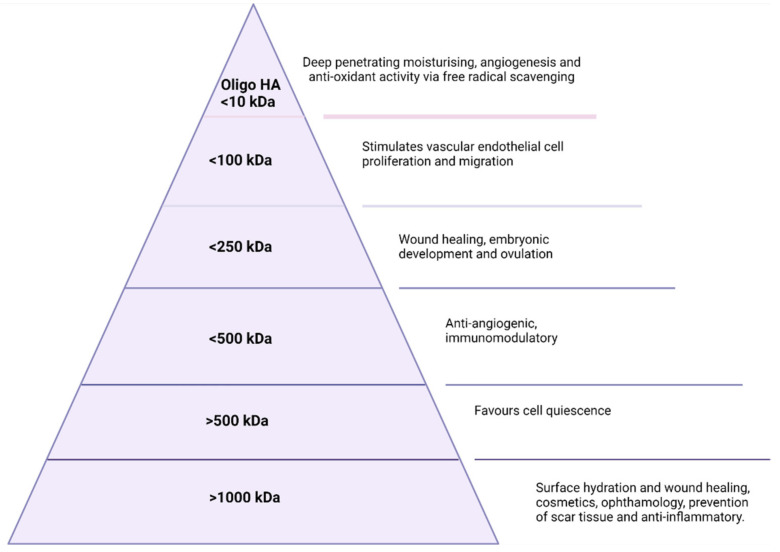
Molecular weight-dependent applications of HA.

For both commercial and endogenous HA, the applications are dependent on molecular weight as illustrated in Figure 2 below. HA of an MW larger than 1000 kDa is primarily useful in the surface hydration of cells and has applications in ophthalmology, wound healing, and cosmetics [28]. Between 10 kDa and 1000 kDa, HA plays a vital role in wound healing. HA between 100–250 kDa has a role in embryonic development and ovulation and is necessary for successful ovulation and fertilisation in most mammals [29]. Finally, oligosaccharides with an MW of ≤10 kDa are critical in promoting fibroblasts’ proliferation, and angiogenesis, and are also implicated in tumour growth [30,31,32]. As indicated in Figure 2, an MW of less than 10 kDa also finds application in the cosmetics industry as the smaller size allows for deeper penetration and subsequent hydration of the skin layers [33]. It is in the cosmetics industry that the majority of the population has been introduced to the unique physicochemical properties of HA, such as viscosity and lubrication via the extraordinary ability of HA to bind water molecules, which has become a staple ingredient in products such as serums and creams. Additionally, the viscoelastic properties of HA have been utilised in cosmetic procedures such as dermal fillers as the viscoelasticity and biodegradability lend themselves perfectly to a flexible, comfortable, biocompatible, and biodegradable filler material for lips, cheeks, and jaws to name but a few [34]. These fillers serve to replace lost volume and hydration from the skin and consist of cross-linked HA. The crosslinkers utilised depend on the desired physical or biological response sought. The water binding and viscoelastic properties of hyaluronic acid have also been exploited in the area of micro-needles. Micro-needles are medical devices, of approximately a micron in size, which penetrate the outermost layer of skin for the purpose of improving the transport of therapeutic through the epidermis [35,36,37]. Extensive research has been conducted over the past decade in micro-needle based drug delivery, and as a result, HA-based micro-needles are used extensively in both the pharmaceutical and cosmetics industries. In this way, HA is used as a dissolving microneedle, created through processes such as micro-molding which is an economic method suitable for mass production. These HA-based microneedles can facilitate the delivery of a variety of molecules such as adenosine and bioactive proteins for the catalysis of collagen and elastin [35], and alendronate for osteoporosis [36], or insulin for diabetes [37].

## 4. Endogenous Bioactive Properties

### Receptor Interactions

HA interacts with various molecules and receptors and conducts numerous functions throughout the extracellular matrix (ECM) via specific and non-specific interactions. Some of the most commonly known receptors that HA interacts with are Neurocan, the receptor for hyaluronan-mediated motility (RHAMM), GHAP (glial HA binding protein), CD44, Aggrecan, and TSG6 (TNF-stimulated gene 6) [38,39]. The most biologically relevant receptor is CD44 due to its multifunctional cell surface conjugated protein that is present in an abundance of cell varieties. These cell surface binding proteins possess key residues which allow for wrapping around and securing the HA polymer chain to the CD44 receptor.

**Figure 3:**
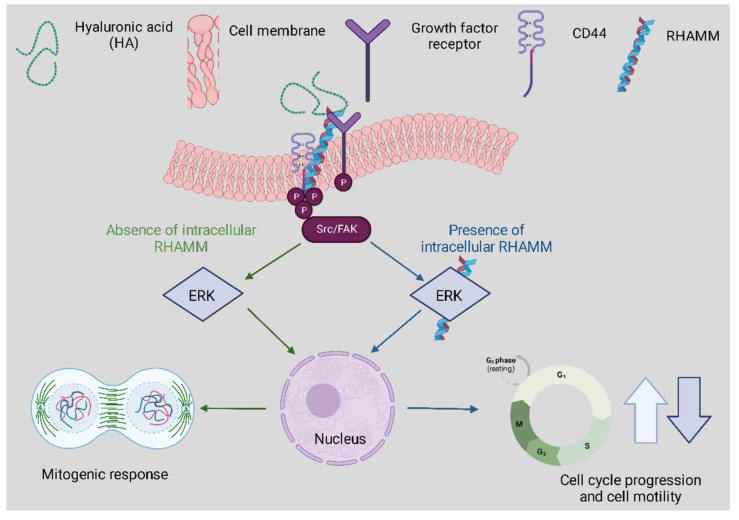
The correspondence between RHAMM and CD44 following HA binding affects physiological and cellular functions. The track denoted in green highlights extracellular signaling involving CD44-HA mediated pathways. The blue track is for intracellular RHAMM signaling. Cell surface RHAMM interacts with CD44, HA, and growth factor receptors (GFR) to activate protein tyrosine kinase signaling cascades that activate the ERK1/2 MAP kinase cascade in a c-Src/FAK/ERK1/2 dependent manner (depicted in green track). In the absence of intracellular RHAMM, this signaling can stimulate the transcription of mitogenic effectors to regulate a mitogenic response (cell proliferation/random motility). In the presence of intracellular RHAMM (blue track), MEK-1/p-ERK1/2 also binds to a number of protein partners that allows activated RHAMM to enter the nucleus to regulate functions of microtubule dynamics via centrosome structure/function, and cell cycle progression. Activated RHAMM also controls the expression of genes involved in cell motility. Overall, the effect of HA is pro-proliferation and the development of cellular infrastructure whilst providing critical immune support.

HA has many functions within tissues correlated to its interactions with the primary receptors CD44 and RHAMM. CD44 expression is a known activation marker that aids in classifying memory and effector T cells. It can also assist in early T cell signaling as it is bound to the lymphocyte-specific protein kinase [39]. CD44 also contributes to cell adhesion interactions and proliferation as illustrated in Figure 3 below above [40]. Despite the binding of HA to CD44, it has been evidenced that HA degradation can trigger inflammation through toll-like receptors such as TLR2 and TLR4 in macrophages and nerve fiber cells [41]. Both TLR and HA are vital components of the innate immune system.

## 5. Synthesis

HA is the only one of the mucopolysaccharides that are not synthesised by the Golgi apparatus. Despite the relatively simple structure of HA, it possesses a range of physiological roles in humans and animals. HA can be isolated from animal sources such as rooster comb, or certain bacteria such as Streptococcus. However, purification from sources such as these is difficult due to the inherent variability with animals and the presence of endotoxins in Streptococcus species. The main sources of commercial HA for the industry are either animal or microorganism derived.

### 5.1. Microbial Synthesis

**Figure 4:**
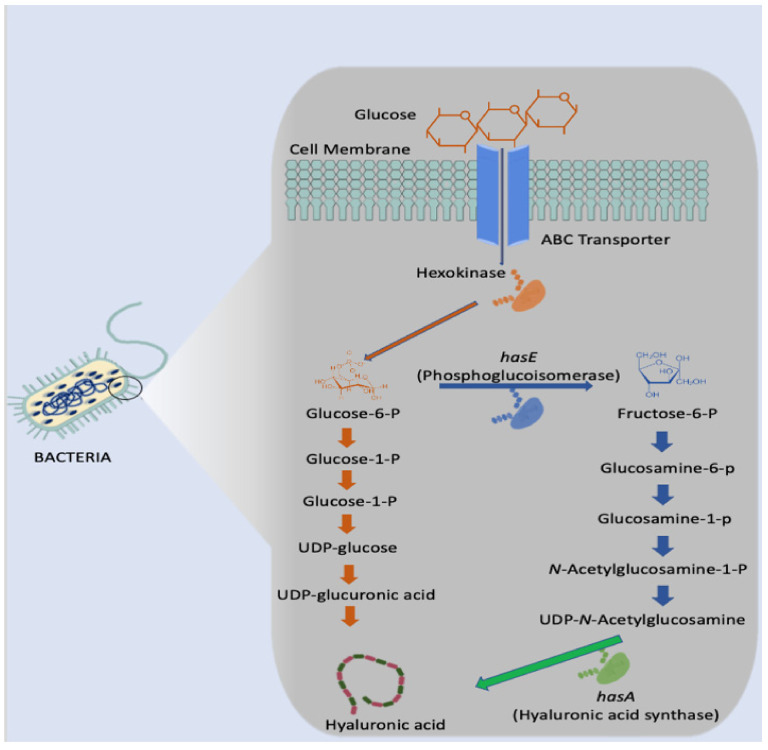
Microbial synthesis of hyaluronic acid in *Streptococcus*.

For bacteria, such as the Streptococcus genus, three distinct genes are required to synthesise HA- HasA, HasB, and HasC. In the initial stage of HA biosynthesis, glucose is converted to glucose-6-phosphate via the enzyme hexokinase as illustrated in Figure 4 below. Glucose-6-phosphate is the most vital precursor in this biosynthesis pathway. Following this initial stage, there are two distinct routes in which two building blocks are produced: glucuronic acid and N-acetyl glucosamine [42,43,44,45]. However, Streptococcus are renowned for producing several endotoxins which would render the HA produced unsuitable for human use. Additionally, the expensive growth media necessary and difficulty in controlling the fermentation process make this genus less than ideal [4].

To overcome the issues with Streptococcus production of HA, researchers have been investigating other strains which are generally regarded as safe (GRAS) and engineering them for HA production. One such strain is Lactococcus lactis, which was engineered by Sheng et al. using the HA biosynthesis operon and the lacF selectable marker [46].

### 5.2. Animal Synthesis

**Figure 5:**
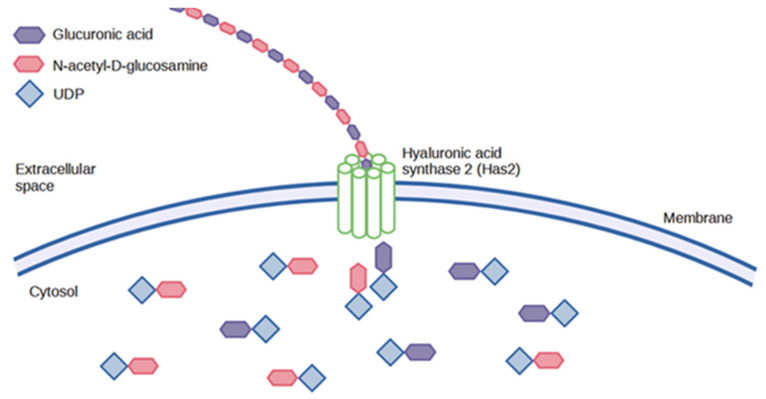
Animal synthesis of HA.

In vertebrates, there are three different has isozymes- has1, has2, and has3, which are involved in HA synthesis during embryonic development [47], morphogenesis [48], wound healing [49], aging and cancer progression [49]. The function of HA synthases is to lengthen the polysaccharide by repeated addition of glucuronic acid and N-acetyl-D-glucosamine groups as illustrated in Figure 5 below. These are then extruded into the cells through the cell wall via ABC-transporters [50]. The different forms of has proteins possess other kinetic profiles, which ultimately affect the size of the HA produced. Has1 and has2 proteins are moderately active and implicated in the synthesis of high MW HA, whereas has3 proteins are highly active and produce low MW HA [38].

HA extracted from animal tissues such as rooster comb still remains an important product due to the high molecular weights which can be recovered when isolating HA from animals. However, the harsh extraction process often results in poor yield and polydispersity of molecular weights [51]. This is due to the grinding, acid treatments, and organic extractions which are necessary to extract the polysaccharide. Additionally, contaminant proteins are a significant issue in the isolation of HA from animals. Cellular proteins such as hyaluronidase, a HA-specific enzyme, may be bound to the polymer in animal tissues and could potentially elicit an immune response in humans if not completely removed from the end product. Similarly, there is the potential for nucleic acid contamination or the spread of animal prions which could result in infectious disease spread [52]. Therefore, the molecular weight advantage of animal extraction is offset by the high cost and labour-intensive processes involved. Thus, biotechnological solutions are the preferred route of commercial HA synthesis where possible.

## 6. Degradation

Endogenous human HA is primarily degraded by an enzyme family known as hyaluronidases (HYAL, but may also be initiated by free radical degeneration. Free radical degradation is a process by which free radicals, or pro-oxidants, cause oxidative stress resulting in organic damage to molecules or cells. Free radical degradation, in particular, has been implicated in HA degradation in aging and arthritis. It initiates degradation via non-specific scission of the glycosidic bond [53], and the concentration of free radicals is directly proportional to the degree of degradation of the HA molecule. The half-life of HA in human tissue ranges from three to five minutes in blood to approximately 70 days in the eye’s vitreous body [24,54]. This turnover rate is controlled by localised degradation or uptake and hydrolysis via the lymph system.

Extracellularly, there are various ways in which larger molecules of HA can be degraded into smaller fragments. This is important as fragmented HA can be used as an indicator of early disease in conditions such as arthritis, or a selection of molecular weights for specific applications may be needed as detailed earlier. For instance, smaller HA fragments are preferred for use in cancer treatments, as an antioxidant, or in cosmetics whereas medium chain HA can be useful in wound repair and regeneration [55,56]. External or extracellular methods include chemical degradation, physical force, free-radical cleavage, pH, temperature, ultrasonic stresses, and, of course, enzymatic degradation.

### Enzymatic Degradation

Enzymatic degradation is performed by HYAL, of which six have been identified in humans- HYAL1, HYAL2, HYAL3, HYAL4, PH20, and HYALP1 [57]. The function of the hyaluronidases is to cleave the large molecule into smaller oligosaccharides. In contrast, β-D-glucuronidase and β-N-acetyl hexosaminidase further degrade the fragments by removing non-reducing sugars from the terminal ends [9]. The oligosaccharides and very low molecular weight fragments produced by this enzymatic degradation have exhibited angiogenic properties in numerous studies. They have also been identified in the disease process of degenerative diseases such as arthritis [58,59,60]. This is in stark contrast to the anti-angiogenic and anti-inflammatory properties displayed by high molecular weight (HMW) HA.

The small fragments of HA modulate gene expression in many cell types. They could invoke an inflammatory response through interaction with toll-like receptors (TLR) such as TLR-2, TLR-4, and CD44, which induce NF-kB activation that, in turn, is responsible for inflammatory mediator transcription such as TNF-α and Il-1β [61]. Despite this, there is growing research into using these oligosaccharides of HA (<10 kDa) to modulate the inflammatory response. Wang et al. demonstrated how these oligos could be used as an agent for reconstructing cardiac function against myocardial infarction [62].

This fragmentation of HA also interferes with HA signaling. There is a working hypothesis that HMW HA can cluster receptors on the cell membrane. In contrast, low molecular weight (LMW) cannot gather the cell membrane proteins the same way; therefore, signaling modulation differs from that induced by HMW in the same cells [63]. Thus, the signaling capabilities of HA rely heavily upon fragmentation.

## 7. Modification to Improve Functionality

Native HA has found a broad range of applications in areas such as ophthalmology and cosmetics due to its unique physicochemical characteristics. To further expand the applications of this polysaccharide, it can be modified to allow for cross-linking and engineering, to tailor the degradation profile in vivo, to improve cell attachment, or to enable conjugation. 

**Figure 6:**
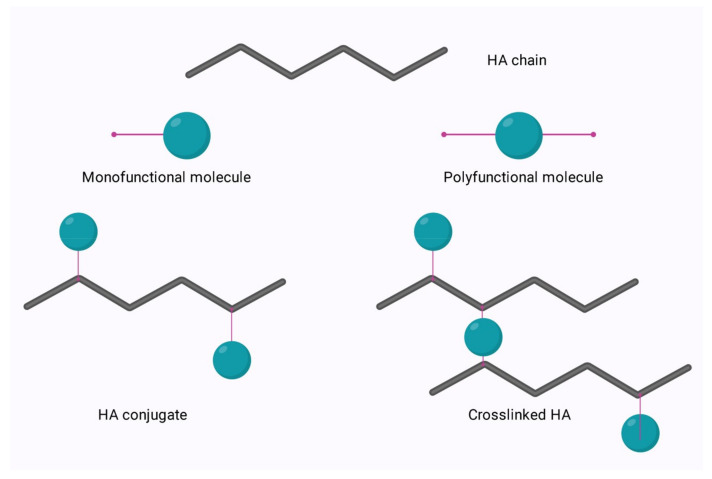
Conjugation and crosslinking of HA.

The relatively simple structure of HA allows for ease of modification of its two main functional groups- the hydroxyl and the carboxyl groups. Additionally, further synthetic modifications may be performed following the deacetylation of the acetamide group, which can allow for the recovery of amino functionalities [64]. Regardless of the functional group to be modified, there are two options for modification; crosslinking or conjugation. These options are shown in Figure 6 below.

Conjugation is modification via the grafting of a molecule onto the HA chain by a covalent bond, whereas crosslinking involves the formation of a matrix of polyfunctional compounds which link chains of native or conjugated HA via two or more covalent bonds [65,66]. Crosslinking can be performed on either native HA or conjugated HA. This is of particular interest in the area of bioconjugation.

Bioconjugation is the act of conjugating peptides or proteins to a natural polymer to increase efficacy. Previously, this was performed using polyethene glycol (PEG). PEGylation was found to increase the effectiveness of drugs by reducing renal clearance, enzymatic degradation, and immunogenicity in vivo. However, repeated injection of PEGylated liposomes has been found to cause accelerated blood clearance and trigger hypersensitivity [67]. Thus, HA is now under investigation as a plausible alternative [68].

Conjugation allows for crosslinking with a variety of molecules to enable the improvement of drug carrier systems with optimised properties. The crosslinking of HA allows for fine-tuning of many characteristics, such as mechanical, rheological, and swelling properties, and protects the polymer from enzymatic degradation to allow for longer residence time at the required treatment site. The process of bioconjugation and crosslinking has found applications in medicine, aesthetics, and bioengineering to treat various ailments. The different approaches and applications of functionalisation have been discussed in great detail by Sanjay Tiwari and Pratap Bahadur (2019) [69], so only a brief overview of hydroxyl and carboxyl group chemical modifications will be discussed in this review.

### 7.1. Modification of HA via the Hydroxyl Group

The standard recognition by degradative enzymes is preserved by retaining the carboxyl group and modifying the hydroxyl groups. Each disaccharide unit of HA consists of four hydroxyl groups, one amide group, and one carboxyl group. One of the most highly marketed HA derivatives, butanediol-diglycidyl ether (BDDE) HA, is produced in an alkaline aqueous solution through simple synthetic procedures [69]. Additionally, divinyl sulfone (DVS) or ethylene sulfide can be used to form other ether derivatives in water [70].

A novel HA drug delivery system targeting tumour cells was created when performing a dimethylaminopyridine (DMAP)-catalysed esterification reaction between butyric anhydride and LMW sodium hyaluronate in dimethylformamide (DMF). Butyric acid has been well reported as an inducer of cell differentiation and inhibitor of various human tumour cells [71]. Other modification methods involve isourea coupling and periodate oxidations. However, both of these methods are performed in harsh conditions and may compromise the integrity and biocompatibility of the HA.

### 7.2. Modification of HA via the Carboxyl Group

The main modifications of the carboxylic group of HA are esterification, carbodiimide mediated, 1-ethyl-3-N, N-dimethylaminopropyl]-carbodiimide (EDC)/N-hydroxy succinimide (NHS) modification, EDC/hydrazide modification and finally thiol modification [72]. HA modified via esterification is usually performed by preparing quaternary salt of HA followed by a reaction with an esterifying reagent. The higher the degree of esterification obtained, the more insoluble the resulting derivative becomes. Two of the best characterised esterified HA derivatives are ethyl and benzyl esters of HA, named HYAFF^®^ 7 and HYAFF^®^ 11, respectively [73,74]. These derivatives were created for tissue engineering applications.

Another option is carbodiimide-mediated modifications whereby the carbodiimide activates the carboxyl group of the HA under acidic conditions. This activation allows for nucleophilic attack of the carboxylate anion to produce O-acylisourea, which the nucleophiles can capture. The most common nucleophilic agents are primary amines despite the low percentage in the nucleophilic amine state at equilibrium [74]. One of the biggest pitfalls of this method is forming the stable intermediate N-acyl urea from O-acylisourea, which can happen in seconds with viscous macromolecules, thus out-competing the exogenous amines [75].

To combat this, a two-step procedure utilising EDC and NHS was created, which was more efficient and increased the yield of modified products. However, the degree of substitution is poor, generally below 20%. This is preferable to most biological investigations so as not to interfere with CD44 interaction [76,77,78,79]. Following chemical conjugation, various crosslinking methods can be employed to allow for use in multiple applications, from tissue engineering to wound healing and aesthetics. Modifications of the carboxylic acid group are summarized below. 

### 7.3. Amidation

**Figure 7:**
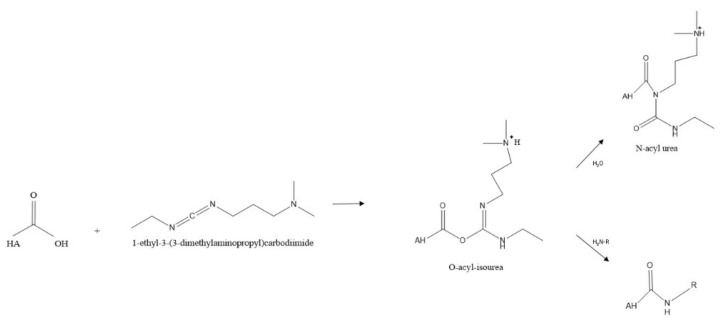
A typical scheme for amidation reaction of HA.

As previously mentioned, carbodiimide modifications are one of the most common modifications performed, typically using EDC due to its water solubility. In this reaction using EDC, the carboxylic acid moieties are activated by EDC, which forms an O-acyl isourea intermediate. In the second step of the reaction, a nucleophilic attack of the amine to the activated HA occurs, forming an amide bond as shown in Figure 7 below. However, the formation of the stable N-acyl urea by-product may occur due to the reaction of the O-acyl isourea with water [67]. If this occurs, no further reaction with the amine takes place. For this reason, many researchers use catalysts such as 4-dimethylaminopyridine (DMAP) in an attempt to push the reaction forward and reduce the amount of N-acyl urea formed [80]. 

Other variations of this carbodiimide-mediated amidation exist also, such as using biscarbodiimides as the reacting reagent itself rather than just an activator or adding NHS to prevent the formation of the stable unreactive by-product N-acyl urea, all of these have been discussed in detail by Schanté et al., 2011 [67]. 

### 7.4. Esterification

**Figure 8:**
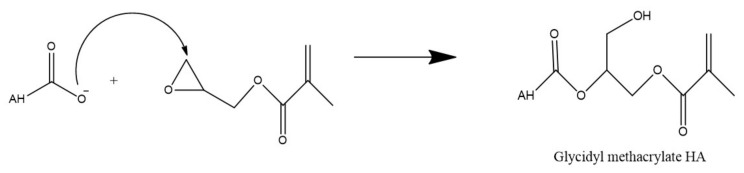
Esterification of HA via glycidyl methacrylate.

Alternatively, the carboxyl group of HA can undergo esterification via a variety of methods such as using alkyl halides, tosylate activation, diazomethane, or epoxides. As these methods have been described in detail by Schanté et al., 2011 [67] and Huang and Chen, 2019 [81], only ester formation via epoxides as shown in Figure 8 below, will be illustrated in this review. 

This reaction is performed in water and in this example, utilizes triethylamine as a catalyst to synthesize methacrylated HA from glycidyl methacrylate. Due to the high reactivity of the methacrylate and the many functional groups of HA, there is some concern regarding the specificity of this type of reaction. However, Bencherif et al. (2008) suggest that the reaction primarily occurs at the carboxylic acid but any esterification which does occur at the hydroxyl groups is reversible [82].

## 8. Immunomodulation Properties

The principal function of the immune system is defence, either against foreign matter, including pathogens or against disease, including cancer [83]. The immune system’s complexity occurs when the immune response either fails to respond to a pathogen or is over-exasperated. Interventions such as vaccines can improve response. Steroids or anti-inflammatory medications can reduce hyper-inflammation. Inflammation is a key, protective immunological function. If not appropriately controlled, it can cause harm to the host and lead to pathologies. Inflammation is linked to several chronic diseases. With increasing numbers of autoimmune conditions and infectious agents, molecules that interact positively with the immune system are always in demand [84].

Bioactives are molecules that can interact with the immune system, in particular cells of first-line defence, including monocytes or macrophages. Bioactives with immune-modulatory activity are of particular interest as they can reduce inflammation without affecting pathogen clearance. Physiochemical properties of bioactives are correlated to activity. These properties include; the final form of the polymer (3D printed, hydrogel, or solid), crosslinking density, and whether the material is synthetic or natural. It has been reported that a high crosslinking density of biomaterials can promote inflammatory macrophage responses [85,86]. In contrast, the opposite appears to be true in the case of HA [87]. 

Immunity can be roughly categorised as innate or nonspecific or acquired or specific. Innate immunity, a rapid response is the earliest line of defence against nonspecific invaders. Included under the innate system are monocytes, macrophages, dendritic cells, and neutrophils. Acquired immunity is a slower response that develops after the initial exposure and is reliant on B- and T-cells. After the initial exposure, the secondary response is rapid and specific. Immune cells, both innate and adaptive, are integrated as they communicate with each other through soluble mediators [88].

### 8.1. The Role of Hyaluronic Acid in Inflammation

HA is a significant component of the extracellular matrix (ECM), which becomes fragmented during infection and tissue injury and is repaired when inflammation subsides. During inflammation, HA turnover is disrupted and HA fragments collect extracellularly. These fragments are linked to the proliferation of the inflammatory response, whereas the full-length, high molecular mass HA is linked to the resolution of inflammation. While all immune cells express the HA receptor CD44, under homeostatic circumstances only a few bind HA. This, however, is altered when immune cells are activated [89].

In response to changes in cell sensitivity and signaling pathway regulation, the expression levels of inflammatory genes are modulated by complex mechanisms. Most likely attributed to hyaluronidase activity, the chain length of high-molecular-weight HA reduces during inflammation [89]. HA absorption and fragmentation by macrophages may reduce inflammation [89].

Toll-like receptors are a family of pattern recognition receptors, which distinguish specific structures in pathogens. Plasma membranes express extracellular TLRs (TLR1, TLR2, TLR4, TLR5, TLR6, and TLR10). Through their extracellular/luminar leucine-rich repeats (LRRs) and cytosolic toll-like/interleukin-1 receptors, these molecules detect infection-derived ligands (TIR). TLRs are expressed on macrophages, neutrophils, dendritic cells (DCs), natural killer (NK) cells, mast cells, T and B lymphocytes, stromal cells, and tumour cells [90].

Both low and high molecular weight HA stimulates TLR-4. Conversely, LMW HA induces the activation of the NF-κB pathway which is associated with inflammation. HMW HA prevents lipopolysaccharide (LPS) a bacterial endotoxin, and activation of macrophages which is anti-inflammatory activity [91]. This contrasting activity demonstrates that the molecular weight of HA has a huge influence on the mode of action and ultimately response.

### 8.2. The Importance of Molecular Weight in HA Immunomodulation

Considering the importance of physicochemical properties in relation to bioactivity, it is surprising that there are currently very few investigations of the immunological responses induced by HA of varying molecular weights. Studies have demonstrated that antiangiogenic, immunosuppressive, and anti-inflammatory properties are seen in HA with a molecular weight larger than 1000 kDa. In contrast, pro-inflammatory, pro-angiogenic, and immunostimulatory characteristics are seen in medium- and low-molecular-weight HA [92].

An interesting study by Lee et al. (2021) tested HA at molecular weights of 10 to 1500 kDa and concentrations of 10 and 100 µg/mL on LPS-stimulated macrophages which are essentially inflamed [91]. They tested these parameters for the pro and anti-inflammatory effects of HA. Nitric Oxide (NO) generation from LPS-stimulated macrophages was used to measure HA-induced inflammation. They also evaluated the impact of different molecular weights of HA on M1 (Inflammatory) and M2 (anti-inflammatory) polarisation of macrophages. They also measured pro- and anti-inflammatory gene expression. Results demonstrated that various molecular weights of HA have distinct effects. LPS-unstimulated and LPS-stimulated macrophages exhibited differential regulation of inflammatory mediators, including cytokines and chemokines, based on the HA molecular weight. In the NO experiment with LPS-stimulated macrophages, HA demonstrated molecular weight-dependent effects on macrophages. Low molecular weight HA (50 kDa) increases iNOS levels significantly in LPS-stimulated chondrocytes. HA with a molecular weight of 1000 kDa had no noticeable effect on iNOS in LPS-stimulated chondrocytes. HA with a high molecular weight (5000 kDa) effectively decreases the iNOS increase generated by LPS.

In addition, they evaluated the impact of various HA molecular weights on the expression levels of certain immune gene expression levels in LPS-unstimulated and LPS-stimulated macrophages. In response to LPS, macrophages will secrete inflammatory mediators, such as IL-6 and TNF-α. Macrophages treated concurrently with LPS and HA have the opposite effect, with TNF-α expression levels decreasing. Il-10 is a cytokine associated with anti-inflammatory pathways. In unstimulated macrophages, HMW HA significantly up-regulated IL-10 compared to other conditions tested again demonstrating that HMW HA influences an anti-inflammatory phenotype [90].

### 8.3. Immunomodulatory Applications of HA

There is always a need to enhance the immune-boosting capabilities of vaccinations. Efforts are being focused on enhancing the immunogenicity of viral vaccines through the use of bio-adhesive delivery systems containing natural ingredients in order to counteract the negative side effects of conventional adjuvants. These bio adhesives adhere to the surfaces of mucosal cells via receptor-mediated processes. HA is one of these natural bio-adhesives that has the ability to stimulate an effective immune response.

In a study β-propiolactone (βPL), binary ethyleneimine (BEI), and hydrogen peroxide (H2O2) were assessed for their inactivation potentials. Monitoring the humoral and cellular immune response induced by rabies vaccinations adjuvanted with Staphylococcus aureus-derived HA and BCG pure protein derivative (PPD). Results showed that both adjuvants may boost the release of anti-rabies total immunoglobulin G and pro-inflammatory mediators in a progressive manner. HA adjuvanted rabies vaccination produced a greater immunological response compared to PPD adjuvanted rabies vaccine [93]. This further demonstrates that HA has a significant influence over immune response.

Mortality and morbidity linked with chronic obstructive pulmonary disease (COPD) are on the rise globally. HA with a high molecular weight is a physiological component of the lung extracellular matrix and possesses considerable anti-inflammatory and hydration capabilities. Therefore, HA was administered in a pilot study of 41 patients. Significantly shorter durations of non-invasive positive-pressure breathing (NIPPV) were seen in patients treated with HA. Additionally, HA-treated individuals had reduced ventilator-measured peak airway pressures and lower systemic inflammatory biomarkers. HA considerably increased mucociliary transport in air–liquid interface cultures of primary bronchial cells from COPD patients and healthy primary cells exposed to cigarette smoke extract, based on simultaneous in-vitro assays [94].

Collectively, the research studying the impact of HA on immune system disorders, namely inflammation, reveal that the molecular weight of the chemical is crucial. HA with a greater molecular weight appears to have anti-inflammatory effects that might be beneficial for a variety of inflammatory illnesses and needs to be explored further.

## 9. Medical Interventions

**Figure 9:**
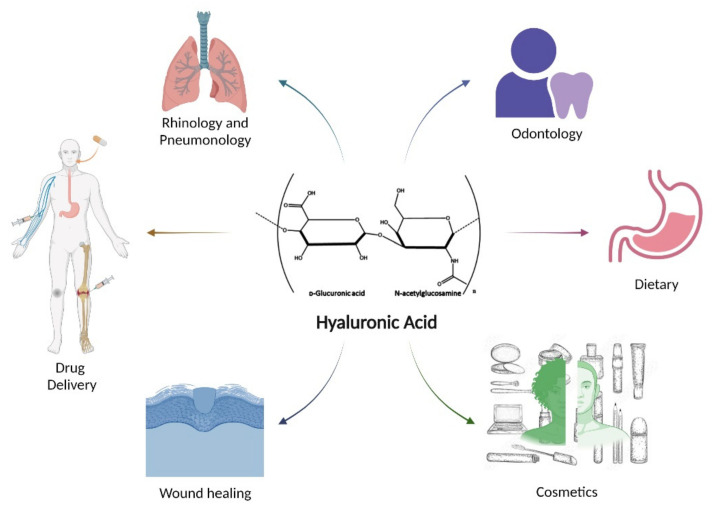
Some of the many applications which utilize hyaluronic acid or HA derivatives.

Through modification and functionalisation of this ubiquitous polymer, a broad range of applications are possible as outlined in Figure 9 below. This section will discuss just some of the many uses HA has found in areas of cancer drug delivery, topical drug delivery, wound healing, inflammatory arthritis, and others.

### 9.1. Targeted Drug Delivery of Cancer Therapeutics

The use of HA in drug delivery is a relatively new concept. It hinges on the actions of receptors such as CD44 and RHAMM, which are responsible for the receptor-mediated endocytosis of HA in vivo. This is particularly true in the case of cancer treatments, where it has been shown that the cellular uptake and efficacy of tumour-targeted drugs are increased when using HA delivery systems [95].

The targeted delivery of anticancer drugs is paramount given the indiscriminate detrimental effects of the compounds on healthy and cancer cells. Efficient targeting of these drugs allows for concentrated dosing at particular sites whilst preserving the surrounding environment. CD44 activity is vital as a variant of this receptor (CD44v) is overexpressed on the surface of many cancer cells, such as epithelial tumour cells, and specifically binds HA [96]. This variant has been shown to have a higher affinity for HA than the standard CD44 receptor (CD44s) [97,98].

This specificity allows for the function in targeted drug delivery of HA and its derivatives, which can carry proteins, peptides, nucleic acids, and various anticancer drugs [99]. This action was demonstrated by using HA decorated liposomes via aminooxy coupling reaction by Bartheldyová et al. (2018). The cells with upregulated expression of the CD44 receptors showed more significant interaction with the HA decorated liposomes, showing promise for tumour targeting in the future [100].

HA can also be directly conjugated to a drug for delivery through endocytosis mediated by the CD44 receptor, as shown by the conjugation of HA to paclitaxel, a taxane with substantial toxicity issues but one which has been proven to improve patient outcomes with squamous cell carcinoma [96]. This conjugation modulates toxicity issues and inhibits the anti-angiogenic effects of taxanes.

### 9.2. Topical Drug Delivery for the Treatment of Skin Disorders

Alternatives to traditional steroid treatments for a variety of skin disorders have been a key area of research in recent years. HA has been used as a topical drug delivery system to treat various skin disorders. This route of administration has numerous benefits over systemic therapies, including avoiding hepatic first-pass metabolism and improved patient compliance. One marketed product of interest is Solarez^®^, a 3% diclofenac in 2.5% HA gel for the topical treatment of actinic keratosis (AK), the third most common skin complaint in the US [101,102]. AK has become synonymous with squamous cell carcinoma (SCC) as the progression of AK into SCC is up to 16% per year on average [99,103], with some dermatologists considering AK as SCC in situ [104]. 

Although the mode of action remains generally unclear, the presence of HA in the topical formula enables deeper penetration of the drug and limits systemic absorption due to the reservation of compounds within the epidermis. This effect is beneficial in treating skin cancer when the use of highly cytotoxic drugs is indicated [105].

### 9.3. Topical Administration for Wound Healing

HA has also found significant use in wound repair, exploiting the physical characteristics of this natural polysaccharide. Following an injury to the skin, HA is expressed within the margin of the wound and is bound with CD44 for keratinocyte migration. HA most likely has a significant role in mediating other wound healing processes such as inflammation, granulation formation, re-epithelialisation, and transformation [106]. 

Endogenous HA can be found at each step of the wound healing process, and HAs effect depends on the MW. LMW HA fragments have been implicated in triggering an inflammatory response due to the affinity of the pieces for the CD44 receptor. This interaction then triggers the activation of macrophages, pro-inflammatory cytokines, and chemokines [107]. Hyalofill^®^ is a wound care treatment that utilises an HA ester derivative mentioned earlier, HYAFF^®^. When this conformable fibrous fleece (Hyalofill-F) or rope (Hyalofill-R) comes into contact with exudate from the wound, it converts into a gel which maintains a moist environment necessary for the formulation of granulation tissue [108]. This product highlights the uses of exogenous HA in wound repair.

### 9.4. Therapeutic Applications in Inflammatory Arthritis

HA has contradictory roles in inflammation, with HMW HA being regarded as anti-inflammatory and oligo-HA or very LMW HA being regarded as pro-inflammatory. Therefore, HA cannot be considered as simply pro or anti-inflammatory. Instead, it is capable of modulating inflammatory responses, which can stabilise the connective tissue matrix [109]. 

This moderating response has been used to treat degenerative knee arthritis via intra-articular injections into the joint. Products such as Orthovisc^®^ and Healon^®^ are used for lubrication and shock absorbing of the joints and have been shown to significantly reduce pain and inflammation in the case of osteoarthritis [110]. Another instance where HA has been used to treat arthritis is by Yang et al. (2019). They demonstrated the efficacy of triterpene, a phytomedicine that could be enhanced by conjugation to HA functionalised bilosomes administered intra-articularly [111]. In this instance, HA improved the circulation and bioavailability of the drug.

### 9.5. Other Therapeutic Applications of Hyaluronic Acid

HA has also been shown to possess antioxidant properties. HMW has been shown to protect against reactive oxygen species (ROS) [112]. Ocular HA drops consisting of HMW HA have been prescribed to treat oxidative stress in patients with chronic dry eye [113]. LMW HA has been shown to stimulate vascular endothelial cell proliferation and migration. Some report that LMW HA stimulates the expression of specific signaling molecules such as ezrin, a necessary protein for cellular adhesion. In contrast, HMW HA displays anti-angiogenic properties by inhibiting the proliferation and migration of endothelial cells. However, these pathways are not well understood as a relatively recent study showed that an injection of LMW HA inhibited tumour growth [114] rather than encouraged it as once thought.

## 10. Future Directions

### 10.1. Drug Delivery in Periodontics

Due to the unique properties of HA, researchers have discovered numerous applications for drug delivery via this polysaccharide. One unique prospect is the use of HA membranes for drug delivery, particularly in periodontics. Periodontal disease is a universal issue, with prevalence reported as high as 50% worldwide [115]. It is characterised as a chronic state of inflammation caused by interactions between bacterial biofilm and the host’s immune system. Current treatment methods consist of biofilm and calculus removal via debridement followed by antimicrobial treatments such as chlorhexidine, tetracycline, or metronidazole [116].

However, systemic antibiotic treatment has significantly been criticised due to the ever-increasing battle against antimicrobial resistance (AMR). Because of AMR, a solution for more localised administration of antibiotics is being sought. HA has been shown to have anti-inflammatory properties at high molecular weights and therefore provides a bioactive medium for the local delivery of antibiotics to treat periodontal pockets post-cleaning to prevent periodontal infections. This research is now in clinical trials investigating the use of HA for furcation or bone defects that occur due to periodontal disease [117].

### 10.2. Hyaluronic Acid as Nanocarriers

Another delivery application of HA currently under investigation is the delivery of nanoparticles. Many materials used for nanocarriers are not fit for purpose due to the inherent toxicity associated with these compounds, however, by coating the nanocarrier in a biocompatible and bioactive substance, this toxicity can be shielded to enable the delivery of the device to the targeted tissue. In this way, HA can be utilized for a variety of applications, from HA-drug conjugates which exploit the functional groups of HA, to HA-based micelles, nanoparticles, and HA coatings for nanocarriers. Regardless of the method selected, the common aim is to alleviate or modulate the toxicity associated with the drug. 

One example is the delivery of metallic quantum dots [118,119] which have found excellent applications in biological imaging and tumour visualisation. However, they are very likely to elicit a strong immune response and systemic toxicity. By shielding or modulating the release of these quantum dots, the potential toxicity may be reduced or staggered to reduce systemic damage.

Another application of this same technology is for the delivery of chemotherapy agents for cancer patients to alleviate the systemic toxicity associated with cancer treatment [120]. A clinical trial that has not yet begun recruiting is investigating just this, with a focus on the delivery of paclitaxel, a common chemotherapeutic agent. They have named this drug delivery system ONCOFID-P-B and it is due to undergo, a phase III study in July 2022 [121].

The physicochemical properties of HA also lend themselves to coatings in nanomedicine for a variety of reasons. Firstly, the hydrophilicity of HA enables the formation of a protein repellant shield around the nanocarrier [122]. Additionally, the anionic nature of the polymer enables the interactions of HA with cationic molecules such as polymers, lipids, and surfactants, which ultimately results in nanostructures being formed [123]. The positive charges of cationic nanocarriers have been described to be shielded by coating with HA, either through electrostatic interactions or chemical conjugation, which is vitalfor enhanced biosafety performance [124]. 

### 10.3. Injectable Hyaluronic Acid Hydrogels

Finally, an area that is quickly evolving is the area of injectable HA hydrogels. There have been multiple clinical trials conducted on HA hydrogels in the treatment of osteoarthritis with mixed results [125,126,127], but now the focus is moving to injectable HA drug carriers for sustained delivery of anti-proliferative chemotherapeutic agents for the treatment of inflammatory arthritis. Similar to nanoparticle delivery, toxicity is greatly modulated and restrained to a precise area of treatment. In a study by Gao et al. (2022), sustained drug delivery was achieved over a period of 4 weeks and the results showed a marked decrease in joint size and interleukin-1β levels [128]. Although this method has not yet made it to clinical trials, it can be postulated that this will be the next avenue explored for the treatment of osteoarthritis with HA. 

Injectable HA hydrogels are traditionally macroscopic hydrogels but there are several disadvantages to these macrogels such as the ease in which they may obstruct blood vessels. To overcome such issues, researchers have been looking at microgels, nanogels, and cryogels as a suitable alternative to macroscopic injectable hydrogels.

Microgel forms of HA have various benefits over traditional HA hydrogels such as increased surface area, and enhanced resilience to degradation of water dispersible micro-gels over water soluble HA [129,130]. This enables the use of microgels for the controlled and extended release of a variety of compounds [131]. Individual microgels can be highly cross-linked to impart resistance to degradation and the physical and mechanical properties of the resulting macro product can be fine-tuned via the degree of intermicrogel cross-linking and dimensions of the microgels [132]. Additionally, characteristics such as anti-microbial efficacy can be imparted on the particles by the cross-linking of HA with the desired compound, such as epoxys [133]. One study by Sahiner et al. (2022) illustrated the use of HA cross-linked with metal ions such as Gadolinium (Gd) and Fe(III) to produce a contrast agent for magnetic resonance imaging (MRI). Their study showed improved contrast capability in MRI imaging as well as excellent cytocompatibility when compared with commercial Gd-based contrast agents [134]. 

Nanogels display similar characteristics in that they display longer blood residence time, and enhanced solubility of pharmaceuticals [135]. This makes them ideal carriers for biological molecules or drugs because the encapsulation in HA nanogels provides improved delivery and targeting effects [136]. 

Cryogels are unique in that they are formed at low temperatures, approximately −20 °C, and have been shown to have shape-memory, an important characteristic for injectable hydrogels. They have been used not only for drug delivery, but also for cartilage repair as the highly interconnected pores allow for the unrestricted flow of nutrients and matrix proteins [136]. 

The wide-ranging application of HA is primarily due to the many administration routes that HA allows for and the list is ever-growing. From modulating the cytotoxic effects of systemic drugs to providing lubrication and sustained release, the future is bright for this unique polymer and there is still much to be unearthed.

## 11. Concluding Remarks

HA is a very unique polymer that has demonstrated value in applications ranging from tissue engineering to drug delivery. The biophysical features of HA allow for a wide range of applications since it can be used in hydrogels that imitate the properties of native soft tissues or in the ECM. Because of its inherent propensity to bind water and reside in a hydrated lattice, HA has a high compressive strength in vivo and can provide lubrication to articulating surfaces, as highlighted throughout this review [137].

The abundance of potential active compounds with restrictions such as poor solubility or stability is one of the key drivers for the future of HA, particularly in drug delivery. Polymers such as HA serve as a bridge for these chemicals to overcome their clinical usage constraints [138]. As previously stated, the border between pro-inflammatory and anti-inflammatory HA decided by MW is a fuzzy region with contradictory results. The transport of these active small molecules is one function of low MW HA (>25 kDa, 250 kDa), as the size facilitates passage through tissues. HMW HA continues to be an issue with biological barriers in the body, such as epithelial, enzymatic, or mucosal barriers, however moderate MW HA may be able to pass this barrier [139]. Because of the numerous potentials for post-modification of the HA molecule, such as hydrogels, superporous cryogels [140], nanoparticles, or conjugates, cancer therapies remain a prominent field of research for HA drug delivery. 

The basic structure and properties of HA allow for modulation of these cytotoxic compounds and can prevent widespread toxicity by targeted release, which is of particular importance given the often-severe side effects associated with many cancer therapies, which HA could modulate through active and passive targeting of tumor cells. Furthermore, because CD44 is a critical receptor for the HA molecule, its amplification in tumor cells allows for more targeted drug delivery. However, as with all-natural polysaccharides, there are many obstacles to overcome such as ensuring stability and longevity in the body, high hydrophilicity, and the difficulty in working with large native polysaccharides such as high viscosity at low concentrations. Despite the various possibilities, these impediments are most likely the reason why HA clinical studies are infrequent, and hence there is a market gap to fully utilize HA.

This paper has illustrated the current applications of HA in a variety of domains; however, there are many more possible routes to pursue. Because current research has proven that HA is superior to other natural polysaccharides for drug delivery applications, well-designed studies evaluating intracellular behavior, biocompatibility, and regulatory aspects of HA should be prioritized.

## Figures and Tables

**Table 1:** Summary of the molecular weights of endogenous HA.

Tissue	Concentration (µg/mL)	Molecular Weight (kDa)	References
Umbilical cord	4100	500	[20]
Synovial fluid	1400–3600	6000–7000	[21,22]
Dermis	200–500	>1000	[23,24]
Epidermis	100	>1000	[23,24]
Thoracic lymph	0.2–50	1400	[3,25,26]
Urine (excreted)	0.1–0.3	4–12	[27]

## Data Availability

Data will be made available on request.
